# Self-Trained LMT for Semisupervised Learning

**DOI:** 10.1155/2016/3057481

**Published:** 2015-12-29

**Authors:** Nikos Fazakis, Stamatis Karlos, Sotiris Kotsiantis, Kyriakos Sgarbas

**Affiliations:** ^1^Department of Electrical and Computer Engineering, University of Patras, 26504 Patras, Greece; ^2^Department of Mathematics, University of Patras, 26504 Patras, Greece

## Abstract

The most important asset of semisupervised classification methods is the use of available unlabeled data combined with a clearly smaller set of labeled examples, so as to increase the classification accuracy compared with the default procedure of supervised methods, which on the other hand use only the labeled data during the training phase. Both the absence of automated mechanisms that produce labeled data and the high cost of needed human effort for completing the procedure of labelization in several scientific domains rise the need for semisupervised methods which counterbalance this phenomenon. In this work, a self-trained Logistic Model Trees (LMT) algorithm is presented, which combines the characteristics of Logistic Trees under the scenario of poor available labeled data. We performed an in depth comparison with other well-known semisupervised classification methods on standard benchmark datasets and we finally reached to the point that the presented technique had better accuracy in most cases.

## 1. Introduction

Classification task is an integral part of machine learning algorithms, trying to separate and thereafter match each tested pattern or object into distinct categories or classes. The classes vary according to the application domain of each problem. For example, the classes could represent the different origin among the tested speakers in a Speech Identification problem or different objects at several pictures in various backgrounds in a Pattern Recognition problem.

The default scenario of classification is the supervised, in which all the available labeled data are used in order to build a classification model. Using the information of the labeled data, the trained supervised classification model will assign to each new instance a class label. Unsupervised techniques can be also used for the same problems. The main characteristic of unsupervised techniques is the lack of need for labeled data [1]. However, the lack of the classes downgrades the performance of unsupervised algorithms, respectively. The most recently proposed family of methods is commonly called semisupervised learning (SSL) algorithms and is generated by a direct combination of the previous strategies [2].

Friedhelm and Edmondo [3] proposed in 2014 a categorization of semisupervised learning algorithms. They used the title of Partially Supervised Learning (PSL) for mentioning of these algorithms. They also referred to the phase of training semisupervised algorithms as a weak supervision, since only a part of the whole information is provided. Trying to explain all the new matters that have arisen from PSL task, Friedhelm and Edmondo [3] review the most prominent directions of research that are related to this domain.

Sun [4] reviews theories in order to describe the characteristics of multiview learning. Under this concept, any set of features, or, more generally, any possible information gathered which is related to the dataset, can potentially improve the classification accuracy. Moreover, Triguero et al. [5] made an in depth study of self-labeled techniques, mainly focused on the matter of classification. Based on some specific properties, which seem to be quite representative of and objective for the majority of real applications, they proposed a taxonomy for semisupervised classification (SSC) methods. One of the findings in this work is the shortage of multilearning approaches introduced with self-training method.

In many application domains, the labeling of the training instances requires high cost in labor and/or time [6]. The major asset of semisupervised algorithms is that they overcome the need for collecting and distinguishing large amounts of data in fields like text mining, speech recognition, object detection from images [7], and so forth, allowing application of such methods in a variety of contexts. Moreover, the increased accuracy that is provided by these methods along with the automated learning of most possible patterns from datasets renders semisupervised techniques as a great tool to the machine learning community [8]. Using SSC methods, the essential effort from human experts of labeling instances tends to be reduced dramatically, especially in real-life scenarios [9].

In particular, SSC methods demand only a small proportion of the whole amount of data to be labeled for accomplishing their task. This attribute is widely known as labeled ratio and is usually provided in percentage values:

(1) Labeled  RatioR%=Number  of  labeled  dinstancesNumber  of  all  the  instances.

Having chosen the labeled ratio, all the available data split into two different subsets: the labeled (*L*) and the unlabeled (*U*) set. The mathematic expression of the instances that are included in each of these subsets is as follows:

(2) Dataset's  instances=xL=Feature  set ∣ True  classxU=Feature  set ∣ Not  known  class.

Tanha et al. [10] suggested that using decision tree classifiers as base classifiers along with self-training algorithm is not quite effective as semisupervised learning is concerned mainly due to low performance when decision tree classifiers compute probability estimations for their predictions. However, decision trees are not demanding in training time and produce easily comprehensive models. A series of modifications have been proposed so as to refrain from using the simplistic proportion distribution at the leaves of a pruned decision tree [11]. Laplacian correction and grafted decision trees are some of them [10]. Torgo [12] also made a thorough study of tree-based regression models and focused on generation of tree models and on pruning by tree selection.

The aim of our work was to present a self-trained Logistic Model Tree (LMT) algorithm and compare it with other well-known semisupervised classification methods on standard benchmark datasets. To achieve this, we performed statistical comparisons of the proposed method with other algorithms and represented an illustrative visualization for recording the behavior of each algorithm against the others. Our proposed technique presented higher accuracy in most cases and a better overall performance in different scenarios, rendering this algorithm as a robust tool.

In Section 2, a brief description of the semisupervised classification techniques is provided. In Section 3, the proposed algorithm is presented. In Section 4, there are the results of the comparison of the proposed algorithm with other well-known semisupervised classification methods on standard benchmark datasets. Finally, some conclusion remarks and future research points are presented in Section 5.

## 2. Semisupervised Techniques

Self-training is usually called a wrapper method that constitutes a great tool for semisupervised learning tasks. It is a simple scheme based on four stages [7]. In the first one, a classifier of our choice is chosen and is trained with a small amount of labeled data, which have been chosen randomly from the initial dataset. During the second phase, the classification of unlabeled instances takes place and afterwards a procedure of assessment follows. More specifically, each instance that has achieved a probability value over a defined threshold is considered enough reliable to be added to the training set for the following training phases. Finally, these instances are added to the initial training set, increasing in this way its robustness. All these phases constitute a complete step of the algorithm. Re-training of the classifier is done using the new enlarged training set until stopping criteria are satisfied. Self-training has been proven to perform with great success in many real-life scenarios, even though misclassified instances could occur due to lack of specific assumptions. An important reason why PSL techniques' performance may fluctuate compared with supervised algorithms' performance is the fact that, during the training phase of the former, some of the unlabeled examples will not get labelized, since the termination of the algorithm will have been preceded [3]. This fact means that a part of the total information provided through the dataset will not be exploited under this scheme.

Self-Training with Editing (SETRED) method is a modified approach to self-training proposed by Li and Zhou [13]. Their principal improvement in relation to the basic self-training scheme is the different tackle of misclassified examples which come from the unlabeled set and may incorrectly be merged with the original train set, pushing in this way the performance of the algorithm in inferior level. In order to reduce these occasions, they build a neighborhood graph in *p*-dimensional feature space, whereas *p* is the dimension of the feature vector (*p* × 1). By evaluating a hypothesis test, they finally discard any example whose output of the test was negative.

Cotraining is an equally important scheme that can be considered as a different variant of self-training technique [14]. Its main approach is that the feature space can be exploited with a different way other than combining all its elements. Under this assumption, which keeps up with the multiview learning, cotraining algorithm assumes that, by dividing the feature space into two separate categories, it is more effective to predict the unlabeled instances each time [15]. This assumption seems to be more realistic when the newly formed categories represent a different view of the dataset. Since the cotraining algorithm belongs to the family of self-training schemes, its algorithmic phases are similar to the previously referred ones, under the restriction of the existence of two independent feature vectors for each instance. In the work of Didaci et al. [16], the relation between the performance of cotraining and the size of the labeled training set was examined and their results showed that high performance was achieved even in cases where the algorithm was provided with very few instances per class. However, Du et al. [17], based on an adequate number of experiments, came to the conclusion that relying on small labeled training sets cannot ensure the accuracy of multiview consideration assumptions. In order to exclude the insertion of misclassified instances into the training set at the end of each iteration, several approaches have been proposed. Sun and Jin [18] filtered the predictions of cotraining classifiers with Canonical Correlation Analysis [4]. By applying CCA on paired datasets, the similarities between unlabeled examples of test set and initial train set were calculated in an effective way and only those instances that satisfied CCA's restrictions were inserted into the initial training set.

Wang et al. [19] proposed the usage of some distance metric, which examines the probabilities of belonging to a class between labeled and unlabeled examples. If two examples have the same class probability value, the metric that has been defined by this scenario will boost the example with the smaller distance, to be selected with a higher possibility. Another technique for separating with higher accuracy the predictions of a semisupervised scheme is the combination of more than one classifier. Jiang et al. [20] introduced a hybrid method which combines the predictions of two different types of classifiers for exploiting their different characteristics. The first one is Naive Bayes (NB), which is a generative classifier, and the second is Support Vector Machine (SVM), which is a discriminative classifier. The final prediction is controlled by a parameter which controls the weights between the two classifiers. A review of other similar hybrid methods is also presented in [20]. Moreover, Li and Zhou [6] suggested Co-Forest algorithm, in which a number of Random Trees are trained on bootstrap data from the dataset. As an ensemble method, its behavior is robust even if the number of the available labeled examples is reduced. The principal idea of this algorithm is the assignment of a few unlabeled examples to each Random Tree during the training period. Eventually, the final decision is produced by majority voting. An extension of this algorithm is ADE-Co-Forest which is based on a data editing technique in order to find and reject possibly problematic instances at the end of each iteration [21]. Within its framework, cotraining by committee has been proposed by Hady and Schwenker [22]. Based on the completely known instances of dataset, a starting committee was built. The ensemble methods that were used under this semisupervised scheme were named as CoBag (Bagging), CoAdaBoost (AdaBoost), and CoRSM (random subspace).

RASCO [23] does not consider any specific criterion for splitting the feature vectors, but it implements a random split, so as to train different learners. Following this strategy, the unlabeled data are getting labeled and added to the training set based on the combination of a number of decisions of the learners trained on different attribute splits. Rel-RASCO [24] algorithm instead of random feature subspaces generates relevant random subspaces using relevance scores of features which are obtained using the mutual information between features and class.

Tri-training scheme uses three classifiers using different bootstrap sample of the same dataset to label each unlabeled instance. If two of the three classifiers agree on the categorization of an instance, then this is considered to be labeled and is added to the training set [25]. An improved approach to tri-training scheme is improved tri-training algorithm (im-tri-training) [26], in which some drawbacks of the original model such as unsuitable error estimation, excessively confined restriction, and deficiency of weight for labeled example and unlabeled example were eliminated. The idea of ensemble methods and majority voting has been also endorsed by Zhou and Goldman [27], who proposed democratic colearning. One really interesting asset of this algorithm is the enlarging of the training set of the classifier whose prediction was different with the final one after the voting phase. Sun and Zhang [28] suggested an ensemble of classifiers to be trained from multiple views. Subsequently, only the instances whose classification stemmed from consensus prediction of multiple classifiers are selected as the most confident in order to teach the other ensemble from the new one view.

Huang et al. [29] proposed a classification method based on Local Cluster Centers (CLCC). This algorithm tries to resolve problems that occur when the provided datasets consist of a few labeled training data and facilitates situations in which the labeling process may lead to misclassified instances. Another algorithm which uses self-training scheme is aggregation pheromone density based semisupervised classification (APSSC) algorithm [30]. In this work, the corresponding property was used, as the name of algorithm defines, found in natural behavior of real ants. Actually, it performed well enough and offered promising results for solving real world problems which are related to the classification task. A combination of classifiers under self-training scheme has been proposed by Wang et al. [31]. Their learning approach is named Self-Training Nearest Neighbor Rule using Cut Edges (SNNRCE) and its main advantage is the prevention of problematic examples from being added in each iteration to the initial labeled set through graph-based methods.

## 3. Proposed Algorithm

Our proposed algorithm combines self-training scheme with Logistic Model Tree (LMT) algorithm. A LMT is a decision tree that has linear regression models at its leaves to provide a piecewise linear regression model [34]. As in ordinary decision trees, a test on one of the features is associated with every inner node. For a nominal feature with *n* values, the node has *n* child nodes, and examples are sorted down one of the *n* branches depending on their feature's value. For numerical features, the node has two child nodes and the test consists of comparisons of the feature value with a threshold. The LogitBoost algorithm is used to produce a linear regression model at every node in the tree [35]. The subsets encountered at lower levels in the tree become smaller and smaller; it can be preferable at some point to build a linear logistic model instead of calling the tree growing procedure recursively. There is strong evidence that building trees for very small datasets is usually not a good idea; it is better to use simpler models (like logistic regression) [36]. As for simple decision trees, pruning is an essential part of the LMT algorithm. For LMT, sometimes a single leaf (a tree pruned back to the root) leads to the best generalization performance, which is seldom the case for simple decision trees [11].

Decision trees can generate estimates for the class membership probabilities: the probability for a particular class is just the fraction of the instances in the region which are labeled with that class. In terms of probability estimates, LMT outperforms all other simple decision trees and related algorithms included in the experiments [34]. In this work, we propose a self-training method that uses the power of LMT for semisupervised tasks. The proposed algorithm (self-trained LMT) is presented in Algorithm 1. The self-training process produces good results by using the more accurate class probabilities of LMT model for the unlabeled instances. When fitting the logistic regression functions at a node, LMT has to determine the number of LogitBoost iterations to run. Originally, this number was cross-validated at every node in the tree [34]. To save time, a heuristic that cross-validates the number only once and then uses this number at every node in the tree was used in our implementation. In [37], a similar process was used.

Removal of data points from *U* to *L* is based on estimation of class probabilities. If the probability of the most probable class exceeds the predefined threshold *T*, then this instance is assigned a label. In the proposed algorithm, experimental results that were performed by the authors showed that a good option for the threshold parameter is the value of 0.9, which gave decent results irrespective of the dataset. It was noticed that only a small amount of instances per class in each iteration meets the restriction above.

Algorithm 2 describes briefly the main characteristics of LMT classifier and is focused on the points that distinguish the used classifier from the common decision tree algorithms.

For the implementation, we used the open-source environments of Weka [38] and KEEL [5]. In our implementation, minNumInst was set to 15 and numBoostIter was set to 10.

## 4. Experiments

The experiments are based on standard classification datasets taken from the KEEL-dataset repository [39] covering a wide range of scientific fields. These datasets have been partitioned using the 10-fold cross-validation procedure. For each generated fold, a given algorithm is trained with the examples contained in the rest of folds (training partition) and then tested with the current fold. Each training partition is divided into two parts: labeled and unlabeled examples. In order to study the influence of the amount of labeled data, we examined four different ratios for dividing the training set: 10%, 20%, 30%, and 40%.

Subsequently, we compared the proposed method with other state-of-the-art algorithms into the KEEL tool [39] such as self-training (C45) [7], self-training (SMO) [40], self-training (NN) [32], SETRED [13], cotraining (C45) [14], cotraining (SMO) [41], democratic-co [27], tri-training (C45) [41], tri-training (SMO) [25], tri-training (NN) [41], DE-tri-training (C45), DE-tri-training (SMO) [42], Co-Forest [6], Rasco (C45) [23], CLCC [29], APSSC [30], SNNRCE [31], Rel-Rasco (NB) [24], ADE-Co-Forest [43], cobagging (C45) [22], and cobagging (SMO) [44]. For all tested algorithms, the default parameters of KEEL were used.

The classification accuracy of each tested algorithm using 10%, 20%, 30%, and 40% as labeled ratio is presented in Tables 1, 2, 3, and 4, respectively. The best accuracy value among the different algorithms tested in each experiment is shown in bold style. For our experiments, we used 52 datasets and all the above 22 algorithms, including Self-LMT. The full tables of comparisons can be found in http://www.math.upatras.gr/~sotos/Self_LMT_Results.xlsx.

**Figure 1:**
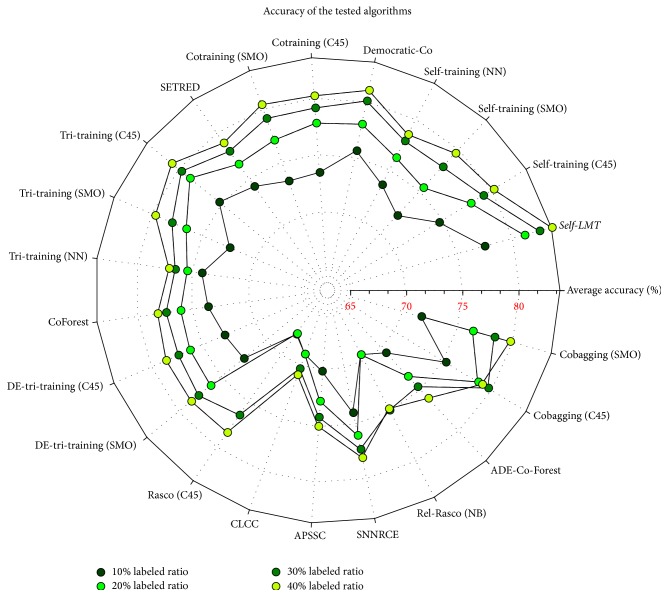
Comparison of average accuracy on benchmark datasets.

Here, we present only the best 10 of these algorithms, according to their classification accuracy. A short comment follows each experiment about the general behavior of the proposed algorithm in comparison with the most effective one of the rest. We also provide a more representative visualization of the average accuracy ability of the proposed algorithm in comparison with the rest 21 algorithms, presented in Figure 1. In this figure, we have mapped each different ratio of labeled instances with a different color across a radar plot.

In this experiment, self-trained LMT and Co-Forest presented 8 wins in an amount of 52 datasets, being followed by self-training (C45), cotraining (C45), and APSSC with 5 victories. Despite the low labeled ratio of instances, self-trained LMT managed to achieve the best average accuracy, assuring its robust behavior.

During the experiment of 20% labeled rate, self-trained LMT algorithm succeeded with 15 victories, while the next in victories' rank were Co-Forest algorithm with 5 and cotraining (SMO) with 4, respectively.

Similar to the previous experiment, self-trained LMT performed 17 wins out of 52 datasets, while cotraining (SMO) and Rel-Rasco (NB) achieved 7 and 6 best accuracy values, respectively.

Finally, self-trained LMT algorithm outperformed the rest of algorithms managing to score the best accuracy value in 19 different datasets, while democratic-co achieved 5 victories.

An interesting point which comes out from Figure 1 is that the increase of labeled ratio does not uniquely mean that the average accuracy of all the algorithms will also be enhanced. The example of cobagging (C45) depicts this phenomenon, since its accuracy rate was decreased when it was provided with 40% labeled ratio against the same rate in 30% labeled ratio scenario. Furthermore, many other algorithms, such as Rel-Rasco (NB), APSSC, and de-tri-training (SMO), did not manage to achieve a noteworthy improvement between 30% and 40% labeled ratio. Consequently, by providing the average accuracy of the tested algorithms on radar plots like this in Figure 1, we can extract useful information for comparing any subset of these algorithms as it concerns not only their accuracy but also their response to labeled ratio's increase, avoiding any saturation phenomena. In order to conduct comparisons among all algorithms considered in the study and the proposed algorithm for all the different labeled ratios, the results of Friedman test together with a post hoc statistical test described in [45] are presented in Tables 5, 6, 7, and 8.

As a result, the proposed algorithm gives statistically better results among all the tested algorithms. This is due to better probability-based ranking and higher classification accuracy which allow selection of the high-confidence predictions in the selection step of self-training.

## 5. Conclusions

It is promising to implement techniques that use both labeled and unlabeled instances in classification tasks. The limited availability of labeled instances makes the learning process difficult, as supervised learning methods cannot produce a learner with worthy accuracy.

LMT produces a single tree containing binary splits on numeric features, multiway splits on categorical ones, and logistic regression models at the leaves, and the algorithm ensures that only relevant features are included in the latter. The produced classifier is not so easy to interpret as a standard decision tree, but much more legible than an ensemble of classifiers or Kernel-based estimators.

In this work, a self-trained LMT algorithm has been proposed. We performed a comparison with other well-known semisupervised learning methods on standard benchmark datasets and the presented technique had better accuracy in most of the tested datasets. Due to the encouraging results obtained from these experiments, one can expect that the proposed technique can be applied to real classification tasks giving slightly better accuracy than the traditional semisupervised approaches.

In spite of these results, no general method will work always. The main drawback of the semisupervised schemes is the needed time in the training phase. Some techniques that could enhance this property by saving both valuable operation time and computational resources are the feature selection algorithms which search for a subset of relevant features by removing the less informative of the initial features [46]. Building Logistic Model Trees with the LMT algorithm are orders of magnitude slower than simple tree induction or using model trees for classification. Improving the computational efficiency of the method using feature selection could be an interesting field for further research.

## Figures and Tables

**Algorithm 1:**
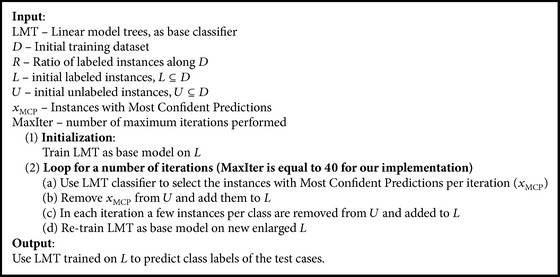
The self-trained LMT algorithm.

**Algorithm 2:**
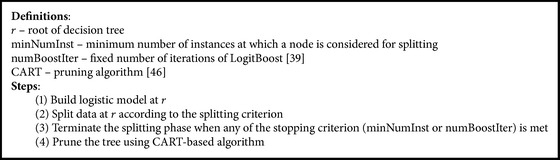
LMT classifier.

**Table 1:** Classification accuracy (labeled ratio 10%).

Datasets	Algorithms									
	Self	Self	Self	CoBag	Cotrain	Cotrain	Democ-Co	Tri-train	Tri-train	SETRED
	(LMT)	(C45)	(NN)	(C45)	(C45)	(SMO)		(C45)	(SMO)	
appendicitis	0.8318	0.8318	0.7573	0.8045	0.8318	0.7927	0.8218	0.8045	0.7527	0.7373
australian	0.8507	0.8275	0.8043	0.8275	0.8348	0.7942	0.8449	0.8449	0.8014	0.8043
automobile	0.3365	0.4052	0.3970	0.3366	0.3730	0.3298	0.3601	0.3889	0.3209	0.4388
banana	0.8547	0.8479	0.8638	0.8553	0.8481	0.8913	0.8417	0.8481	0.8951	0.8638
breast	0.6809	0.7216	0.6618	0.7252	0.6774	0.6696	0.7287	0.7216	0.6973	0.6835
bupa	0.5398	0.5392	0.5314	0.6119	0.5739	0.6123	0.5104	0.5742	0.6291	0.5339
chess	0.9540	0.9543	0.8098	0.9543	0.9515	0.9033	0.9199	**0.9578**	0.9061	0.8104
cleveland	0.5091	0.5248	0.4825	0.5335	**0.5736**	0.5032	0.5223	0.4761	0.5565	0.5294
coil2000	0.9377	0.9373	0.8904	0.9348	**0.9403**	0.6175	0.9322	0.9357	0.5775	0.8926
contraceptive	0.4717	**0.4886**	0.4107	0.4827	0.4460	0.4590	0.4358	0.4813	0.4542	0.4148
crx	0.8555	**0.8660**	0.8111	0.8496	0.8159	0.8177	0.8495	0.8555	0.8393	0.8111
dermatology	0.8627	0.8562	0.9053	0.8760	0.8429	0.7737	0.8760	0.8816	0.6729	**0.9182**
ecoli	0.6883	0.6467	0.6847	0.6558	0.5772	0.6550	0.6370	0.6585	0.6489	**0.6937**
flare	0.7093	**0.7214**	0.6511	0.7140	0.5742	0.5658	**0.7214**	0.7158	0.6587	0.6445
german	0.7140	0.7060	0.6540	0.7110	0.6900	0.6260	0.7160	**0.7170**	0.6610	0.6660
glass	0.5126	0.4847	0.5631	0.4898	0.4504	0.5554	0.4868	0.4921	0.5188	0.5402
haberman	0.7119	0.7054	0.6144	0.7122	0.7190	0.5942	0.7156	0.7088	0.6299	0.6211
heart	0.7333	0.6778	0.7444	0.7037	0.7000	0.7370	0.8000	0.7148	0.6296	0.7444
hepatitis	**0.8365**	0.8343	0.7883	0.8343	0.8343	0.8243	0.8343	0.8343	0.8343	0.8008
housevotes	0.9229	**0.9414**	0.9129	0.9195	0.8225	0.8260	0.8899	0.9158	0.6449	0.9129
iris	0.8400	0.8400	0.9000	0.8000	0.8467	**0.9467**	0.9133	0.7267	0.8933	0.9133
led7digit	0.6620	0.6140	0.6180	0.5640	0.5140	0.5020	0.6160	0.6040	0.5160	0.6180
lymphography	0.6574	0.6312	0.6354	0.5948	0.5726	0.5490	0.4901	0.6118	0.5556	0.6833
magic	0.8363	0.8217	0.7840	0.8319	0.8204	0.8390	0.7842	0.8245	0.8392	0.7840
mammographic	0.8074	0.8025	0.7591	0.8085	0.8074	0.7881	0.7963	**0.8183**	0.7710	0.7580
monk-2	0.9458	**0.9727**	0.6459	0.9657	**0.9727**	0.8146	0.9075	0.9657	0.7571	0.6459
movement_libras	0.3611	0.2556	0.4583	0.2417	0.2361	0.1806	0.1972	0.2750	0.2528	0.4444
mushroom	**0.9968**	0.9966	0.9945	0.9954	**0.9968**	0.9932	0.9927	0.9955	0.9905	0.9945
nursery	**0.9296**	0.9064	0.7143	0.9006	0.9034	0.8141	0.8951	0.9039	0.7714	0.8101
page-blocks	0.9503	0.9523	0.9256	0.9567	0.9492	0.9448	0.9077	0.9561	0.9454	0.9359
penbased	0.9571	0.8916	0.9778	0.9049	0.8957	0.9843	0.9474	0.9027	**0.9855**	0.9778
phoneme	0.7809	0.7770	0.8044	0.7889	0.7652	**0.8320**	0.7874	0.7770	0.8288	0.8046
pima	0.7136	0.6643	0.6565	0.6342	0.6704	0.6395	0.6967	0.6564	0.5990	0.6565
ring	0.8328	0.8396	0.6691	0.8582	0.8366	0.9701	0.8741	0.8542	0.9699	0.6691
saheart	**0.6906**	0.6516	0.6408	0.6497	0.6364	0.5822	0.6819	0.6776	0.6342	0.6300
satimage	0.8340	0.8045	0.8466	0.8205	0.8056	0.8570	0.8462	0.8224	0.8518	0.8570
segment	0.9242	0.8900	0.9061	0.9160	0.9017	**0.9281**	0.9026	0.9000	0.9190	0.9065
sonar	0.7010	0.6433	0.6633	0.7014	0.5819	0.6005	0.6005	0.7019	0.6005	0.6633
spambase	0.8980	0.8669	0.8281	0.8951	0.8884	0.8601	0.8777	0.8810	0.8549	0.8281
spectfheart	0.7085	0.6819	0.6865	0.7571	0.7235	0.7795	0.7379	0.7574	0.7870	0.7201
splice	**0.9345**	0.8266	0.6793	0.8248	0.8310	0.7862	0.8978	0.8254	0.7843	0.6997
texture	0.9805	0.8305	0.9513	0.8500	0.8289	0.9753	0.8944	0.8524	0.9684	0.9513
thyroid	0.9869	0.9922	0.8963	0.9906	**0.9924**	0.9342	0.9393	0.9918	0.9299	0.9090
tic-tac-toe	**0.7557**	0.7108	0.7150	0.7035	0.6931	0.6733	0.6900	0.7088	0.6420	0.7255
titanic	0.7688	0.7751	0.6402	**0.7837**	0.7783	0.7756	0.7756	0.7765	0.7760	0.6402
twonorm	0.9720	0.8136	0.9358	0.8597	0.8085	0.9735	0.9645	0.8616	0.9743	0.9358
vehicle	**0.6526**	0.5792	0.5698	0.6030	0.5747	0.6135	0.5023	0.6194	0.5910	0.5828
vowel	0.4808	0.4242	0.4879	0.4556	0.4384	0.4586	0.4162	0.4525	0.4283	0.4808
wine	0.9157	0.7405	0.9438	0.7866	0.8075	0.9435	0.9493	0.8203	0.8931	0.9438
wisconsin	0.9373	0.9093	0.9478	0.9284	0.9064	0.9592	0.9650	0.9312	0.9563	0.9478
yeast	**0.5304**	0.4616	0.4771	0.4765	0.4886	0.4785	0.4886	0.4907	0.4839	0.4886
zoo	0.8097	0.6794	0.9236	0.7456	0.6356	0.5625	0.9314	0.7192	0.5775	**0.9347**

**Table 2:** Classification accuracy (labeled ratio 20%).

Datasets	Algorithms									
	Self	Self	Self	CoBag	Cotrain	Cotrain	Democ-Co	Tri-train	Tri-train	SETRED
	(LMT)	(C45)	(NN)	(C45)	(C45)	(SMO)		(C45)	(SMO)	
appendicitis	0.8518	0.8509	0.7891	0.8318	0.8500	0.8327	0.8600	0.8318	0.8027	0.8100
australian	**0.8667**	0.8580	0.8304	0.8362	0.8377	0.8304	0.8580	0.8522	0.8435	0.8304
automobile	**0.5523**	0.5158	0.5234	0.5355	0.4803	0.4047	0.4744	0.4938	0.3986	0.4951
banana	0.8685	0.8804	0.8691	0.8804	0.8743	0.8975	0.8794	0.8736	**0.8985**	0.8691
breast	0.7358	0.7156	0.6780	0.7224	0.7227	0.6076	0.7184	0.7225	0.6493	0.6788
bupa	0.6238	0.6055	0.5654	0.6026	0.6055	0.6298	0.5504	0.6199	0.6459	0.5655
chess	0.9778	0.9778	0.8489	0.9778	0.9759	0.9509	0.9393	**0.9781**	0.9459	0.8489
cleveland	0.5462	0.4929	0.4692	0.5535	0.5270	0.4866	0.5530	0.5399	0.5129	0.5257
coil2000	0.9383	0.9400	0.8965	0.9338	0.9402	0.8206	0.9330	0.9371	0.6900	0.8994
contraceptive	**0.5248**	0.4779	0.4324	0.4806	0.5044	0.4793	0.4841	0.4881	0.4705	0.4379
crx	0.8661	0.8571	0.8142	0.8510	0.8498	0.8500	0.8587	0.8540	**0.8700**	0.8142
dermatology	0.9190	0.9100	0.9355	0.9186	0.8790	0.8763	0.9300	0.9214	0.8905	**0.9467**
ecoli	0.7295	0.7204	0.7351	0.7324	0.7329	0.6996	0.7235	0.7208	0.7171	0.7351
flare	0.7251	0.7280	0.6613	0.7176	0.6897	0.6023	**0.7402**	0.7270	0.6332	0.6660
german	0.7110	0.6970	0.6510	0.7120	0.6990	0.6410	**0.7290**	0.6840	0.6580	0.6620
glass	0.5270	0.5085	0.6193	0.5631	0.5348	0.5896	0.4799	0.6104	0.5538	0.5935
haberman	0.7219	0.7089	0.6402	0.7348	**0.7353**	0.6626	0.7222	0.7089	0.6302	0.6600
heart	0.7963	0.7519	0.7741	0.7481	0.7556	0.7778	**0.8222**	0.7926	0.7852	0.7741
hepatitis	0.7848	0.8434	0.7859	0.8200	0.8434	0.8343	0.8343	0.8434	0.8343	0.7992
housevotes	**0.9667**	0.9586	0.8995	0.9528	0.9363	0.8816	0.9023	0.9586	0.8965	0.8957
iris	0.8933	0.8933	0.9067	0.8667	0.8933	0.9400	0.9533	0.8800	0.9200	0.9200
led7digit	0.6840	0.6780	0.6280	0.6580	0.6440	0.6220	0.6620	0.6760	0.6100	0.6280
lymphography	0.7685	0.7055	0.7446	0.7474	0.7750	0.6979	0.4612	0.7545	0.6303	0.7392
magic	0.8513	0.8304	0.7934	0.8409	0.8320	**0.8528**	0.8017	0.8335	0.8451	0.7934
mammographic	0.8232	0.8229	0.7473	**0.8248**	0.8228	0.7968	0.8084	0.8157	0.7907	0.7473
monk-2	**0.9818**	0.9795	0.7346	0.9727	0.9795	0.8960	0.9441	0.9727	0.8800	0.7322
movement_libras	0.4972	0.3472	0.5750	0.3556	0.3917	0.2194	0.3667	0.3833	0.3611	0.6000
mushroom	**0.9995**	0.9991	0.9984	0.9989	0.9991	0.9968	0.9980	0.9989	0.9975	0.9984
nursery	**0.9505**	0.9235	0.7471	0.9235	0.9260	0.8390	0.9108	0.9258	0.7714	0.8327
page-blocks	**0.9618**	0.9602	0.9413	0.9613	0.9609	0.9571	0.9115	0.9611	0.9567	0.9448
penbased	0.9711	0.9241	0.9871	0.9370	0.9247	**0.9916**	0.9632	0.9287	0.9913	0.9871
phoneme	0.8059	0.7839	0.8344	0.8244	0.8024	**0.8497**	0.8055	0.7977	0.8470	0.8344
pima	**0.7553**	0.6810	0.6382	0.7084	0.6874	0.6562	0.7319	0.6939	0.6341	0.6369
ring	0.8520	0.8658	0.7007	0.8918	0.8630	0.9705	0.8969	0.8795	0.9722	0.7007
saheart	0.6907	0.6515	0.6623	0.6862	**0.7038**	0.6041	0.6972	0.6777	0.6256	0.6601
satimage	0.8396	0.8238	0.8702	0.8410	0.8261	0.8693	0.8612	0.8353	0.8707	**0.8740**
segment	0.9407	0.9264	0.9333	0.9277	0.9294	**0.9489**	0.9307	0.9247	0.9364	0.9338
sonar	**0.7736**	0.6636	0.7012	0.6586	0.6336	0.7164	0.6474	0.6631	0.6960	0.7012
spambase	0.9154	0.8912	0.8529	0.8960	0.8908	0.9012	0.8941	0.8941	0.9010	0.8529
spectfheart	0.7719	0.7192	0.6860	0.7833	0.7611	0.6744	0.7305	0.7491	0.7231	0.7085
splice	**0.9417**	0.8834	0.6969	0.8875	0.8793	0.8897	0.9119	0.8843	0.9169	0.6978
texture	**0.9875**	0.8669	0.9713	0.8840	0.8631	0.9865	0.9176	0.8929	0.9842	0.9713
thyroid	0.9939	0.9935	0.9049	**0.9942**	0.9932	0.9403	0.9419	0.9939	0.9392	0.9149
tic-tac-toe	**0.8768**	0.7568	0.7662	0.7286	0.7213	0.7223	0.7329	0.7505	0.7024	0.7621
titanic	0.7833	0.7824	0.6407	0.7828	0.7824	0.7833	0.7797	0.7815	0.7806	0.6407
twonorm	0.9755	0.8165	0.9411	0.8741	0.8276	0.9728	0.9707	0.8674	0.9747	0.9411
vehicle	**0.7141**	0.6489	0.6253	0.6549	0.6489	0.6926	0.4833	0.6620	0.6668	0.6253
vowel	0.6111	0.5303	0.6848	0.5566	0.5303	0.6990	0.5030	0.5545	0.6707	0.6697
wine	0.9265	0.8369	0.9382	0.8255	0.7863	0.9556	0.9542	0.8422	0.9265	0.9154
wisconsin	0.9490	0.9343	0.9478	0.9360	0.9343	0.9490	0.9637	0.9253	0.9503	0.9463
yeast	**0.5728**	0.5263	0.4899	0.5202	0.5492	0.5122	0.5492	0.5486	0.5169	0.5061
zoo	0.8975	0.8311	0.9247	0.8231	0.7344	0.6283	0.8900	0.8264	0.5375	**0.9372**

**Table 3:** Classification accuracy (labeled ratio 30%).

Datasets	Algorithms									
	Self	Self	Self	CoBag	Cotrain	Cotrain	Democ-Co	Tri-train	Tri-train	SETRED
	(LMT)	(C45)	(NN)	(C45)	(C45)	(SMO)		(C45)	(SMO)	
appendicitis	0.8491	0.8409	0.8300	0.8318	0.8409	0.8127	0.8691	0.8327	0.7227	0.8109
australian	**0.8652**	0.8464	0.8101	0.8507	0.8478	0.8232	0.8536	0.8420	0.8116	0.8101
automobile	**0.6631**	0.5484	0.5630	0.5737	0.5710	0.4725	0.5432	0.5707	0.4429	0.5515
banana	0.8796	0.8819	0.8700	0.8811	0.8785	0.8992	0.8728	0.8806	**0.9004**	0.8700
breast	0.7287	0.7179	0.6419	0.7111	0.7079	0.5594	0.7219	0.7000	0.5773	0.6495
bupa	0.6293	0.5842	0.5791	0.6159	0.6137	0.6192	0.5988	0.5850	0.6307	0.5848
chess	**0.9890**	0.9856	0.8651	0.9819	0.9840	0.9628	0.9596	0.9843	0.9512	0.8648
cleveland	**0.5837**	0.5364	0.5159	0.5599	0.5467	0.4733	0.5700	0.5362	0.4619	0.5566
coil2000	0.9390	0.9384	0.8977	0.9317	0.9400	0.8741	0.9320	0.9387	0.6480	0.8992
contraceptive	**0.5132**	0.4922	0.4365	0.5092	0.4976	0.4929	0.4847	0.5105	0.4929	0.4426
crx	0.8568	0.8526	0.8067	0.8615	0.8570	0.8512	0.8482	0.8571	0.8608	0.8067
dermatology	0.9352	0.9209	0.9463	0.9184	0.9098	0.9267	0.9180	0.9240	0.9296	0.9380
ecoli	0.7592	0.7534	0.7593	0.7121	0.7505	0.7116	0.7595	0.7503	0.6910	**0.7652**
flare	0.7392	0.7299	0.6651	0.7308	0.7224	0.6473	**0.7485**	0.7298	0.6623	0.6594
german	0.7370	0.7100	0.6900	0.6920	0.6840	0.6460	**0.7390**	0.7030	0.6610	0.6960
glass	0.5122	0.5437	0.6096	0.5635	0.5746	0.6220	0.5079	0.5687	0.5983	0.5933
haberman	0.7219	0.7153	0.6630	0.7282	0.7288	0.6695	0.7447	0.7089	0.6499	0.6761
heart	0.8000	0.7556	0.7852	0.7704	0.7704	0.7963	**0.8296**	0.7556	0.7778	0.7852
hepatitis	0.7757	0.8192	0.8168	0.8334	0.7934	0.8343	0.8168	0.8334	0.8343	0.8275
housevotes	**0.9702**	0.9475	0.9078	0.9531	0.9531	0.9127	0.9037	0.9531	0.9164	0.9078
iris	0.8933	0.9200	0.9133	0.9067	0.9133	0.9467	**0.9533**	0.9133	0.9467	0.9267
led7digit	**0.7240**	0.6880	0.6360	0.6820	0.6520	0.6760	0.6820	0.6760	0.6780	0.6360
lymphography	0.7430	0.7587	0.7446	0.7445	0.7444	**0.7925**	0.7442	0.7315	0.7858	0.7439
magic	0.8556	0.8380	0.7950	0.8456	0.8385	**0.8562**	0.8016	0.8411	**0.8562**	0.7950
mammographic	0.8282	0.8438	0.7620	0.8352	0.8437	0.8098	0.8300	0.8425	0.7990	0.7620
monk-2	0.9739	**0.9909**	0.7581	0.9886	**0.9909**	0.9030	0.9452	**0.9909**	0.9161	0.7513
movement_libras	0.6333	0.4083	**0.7111**	0.4250	0.4639	0.3833	0.4917	0.4889	0.4583	0.6917
mushroom	**1.0000**	0.9996	0.9991	0.9991	0.9996	0.9984	0.9995	0.9995	0.9988	0.9991
nursery	**0.9578**	0.9377	0.7687	0.9382	0.9363	0.8551	0.9212	0.9362	0.7714	0.8357
page-blocks	0.9645	0.9635	0.9428	0.9622	0.9618	0.9636	0.9289	0.9635	0.9633	0.9461
penbased	0.9757	0.9409	0.9901	0.9485	0.9391	**0.9937**	0.9729	0.9431	0.9908	0.9901
phoneme	0.8229	0.8137	0.8470	0.8275	0.8218	**0.8557**	0.8029	0.8249	0.8534	0.8470
pima	**0.7592**	0.7252	0.6733	0.7085	0.7123	0.6680	0.7305	0.7045	0.6589	0.6694
ring	0.8677	0.8754	0.7104	0.8943	0.8784	0.9708	0.9089	0.8881	0.9722	0.7104
saheart	0.6928	0.6753	0.6644	0.6797	0.6778	0.6039	**0.7080**	0.6797	0.5930	0.6644
satimage	0.8552	0.8270	0.8822	0.8528	0.8407	0.8786	0.8693	0.8438	0.8777	**0.8862**
segment	0.9455	0.9303	0.9411	0.9329	0.9385	**0.9567**	0.9416	0.9381	0.9558	0.9411
sonar	0.7593	0.6762	0.7645	0.7155	0.6333	**0.8264**	0.7310	0.6912	0.7543	0.7645
spambase	0.9195	0.8999	0.8695	0.9117	0.9052	0.9117	0.9052	0.9073	0.9123	0.8695
spectfheart	0.7647	0.7496	0.7010	0.7608	0.7538	0.6859	0.7121	0.7644	0.7046	0.7127
splice	**0.9527**	0.9166	0.7166	0.9201	0.9176	0.9022	0.9188	0.9157	0.9298	0.7154
texture	**0.9905**	0.8891	0.9800	0.9115	0.8971	0.9895	0.9331	0.9055	0.9885	0.9805
thyroid	**0.9956**	0.9946	0.9100	0.9939	0.9947	0.9483	0.9521	0.9944	0.9489	0.9183
tic-tac-toe	**0.9467**	0.7610	0.7975	0.7745	0.7495	0.7755	0.7630	0.7641	0.7233	0.7923
titanic	**0.7860**	0.7787	0.6407	0.7797	0.7783	**0.7860**	0.7792	0.7783	**0.7860**	0.6407
twonorm	0.9746	0.8255	0.9439	0.8778	0.8323	0.9731	0.9701	0.8743	0.9734	0.9439
vehicle	**0.7874**	0.6584	0.6630	0.6561	0.6656	0.7175	0.5652	0.6702	0.7245	0.6583
vowel	0.7263	0.6232	0.7889	0.6172	0.5859	**0.8030**	0.5960	0.6333	0.7828	0.7737
wine	0.9438	0.8415	0.9382	0.8817	0.8255	0.9663	0.9660	0.9039	0.9552	0.9275
wisconsin	0.9534	0.9443	0.9535	0.9431	0.9474	0.9621	0.9666	0.9502	0.9459	0.9535
yeast	**0.5843**	0.5209	0.5000	0.5236	0.5358	0.5270	0.5371	0.5405	0.5244	0.5128
zoo	0.8808	0.8231	0.9331	0.7731	0.8256	0.6597	0.9133	0.7781	0.6197	0.9331

**Table 4:** Classification accuracy (labeled ratio 40%).

Datasets	Algorithms									
	Self	Self	Self	CoBag	Cotrain	Cotrain	Democ-Co	Tri-train	Tri-train	SETRED
	(LMT)	(C45)	(NN)	(C45)	(C45)	(SMO)		(C45)	(SMO)	
appendicitis	0.8509	0.8018	0.7545	0.8382	0.8000	0.7727	0.8591	0.7718	0.7827	0.7564
australian	**0.8739**	0.8507	0.8246	0.8449	0.8551	0.8246	0.8420	0.8435	0.8319	0.8246
automobile	**0.6640**	0.5760	0.6038	0.6009	0.6168	0.5844	0.5537	0.6151	0.5515	0.6089
banana	0.8858	0.8828	0.8649	0.8836	0.8785	**0.9015**	0.8811	0.8825	0.8996	0.8649
breast	0.7294	0.7046	0.6529	0.7289	0.7082	0.5497	0.7257	0.7009	0.5870	0.6632
bupa	**0.6724**	0.6546	0.5760	0.6180	0.6002	0.6462	0.6035	0.6570	0.6347	0.5874
chess	**0.9912**	0.9853	0.8795	0.9872	0.9856	0.9728	0.9712	0.9872	0.9668	0.8795
cleveland	0.5597	0.5093	0.5083	0.5627	0.5364	0.4452	0.5290	0.5289	0.4479	0.5586
coil2000	0.9384	0.9401	0.8967	0.9354	0.9395	0.9109	0.9327	0.9399	0.8881	0.8982
contraceptive	**0.5234**	0.5085	0.4413	0.5011	0.4963	0.4908	0.4977	0.5173	0.4868	0.4379
crx	0.8558	0.8465	0.8161	0.8587	0.8556	0.8120	0.8424	0.8527	0.7882	0.8161
dermatology	0.9467	0.9385	0.9493	0.9267	0.9159	0.9439	0.9439	0.9353	0.9324	0.9466
ecoli	0.7711	0.7739	0.7474	0.7476	0.7768	0.7268	**0.7947**	**0.7947**	0.7148	0.7534
flare	**0.7449**	0.7280	0.6688	0.7223	0.7072	0.6511	0.7420	0.7270	0.6501	0.6679
german	0.7380	0.7100	0.6730	0.6970	0.7170	0.6580	**0.7420**	0.7110	0.6580	0.6790
glass	0.5503	0.5722	0.6555	0.6272	0.6038	0.6699	0.5535	0.6718	0.6103	0.6487
haberman	0.7347	0.7186	0.6791	0.7449	0.7385	0.7026	0.7449	0.7218	0.6692	0.6924
heart	0.8333	0.7667	0.7815	0.7926	0.7407	0.8000	0.8259	0.7741	0.8000	0.7741
hepatitis	0.8517	0.8651	0.7934	0.8619	**0.8785**	0.8443	0.8543	0.8675	0.8343	0.8392
housevotes	**0.9702**	0.9586	0.9117	**0.9702**	0.9586	0.9247	0.9164	0.9591	0.9164	0.9117
iris	0.9200	0.9000	0.9267	0.9067	0.9000	0.9400	**0.9733**	0.9067	0.9467	0.9333
led7digit	**0.7260**	0.6840	0.6380	0.6880	0.6740	0.6880	0.6820	0.6740	0.6920	0.6380
lymphography	**0.8166**	0.7725	0.7247	0.7630	0.7653	0.8050	0.6496	0.7382	0.7841	0.7639
magic	0.8565	0.8416	0.7996	0.8532	0.8405	0.8626	0.8111	0.8409	**0.8628**	0.7996
mammographic	0.8390	**0.8452**	0.7486	0.8352	0.8440	0.8001	0.8267	0.8367	0.7929	0.7486
monk-2	**1.0000**	**1.0000**	0.7761	**1.0000**	**1.0000**	0.9142	0.9543	**1.0000**	0.9103	0.7761
movement_libras	0.6944	0.4889	0.7639	0.4889	0.4611	0.5167	0.6222	0.5194	0.5528	0.7500
mushroom	**1.0000**	**1.0000**	0.9998	0.9993	**1.0000**	0.9991	0.9998	0.9995	0.9993	0.9998
nursery	**0.9652**	0.9442	0.7883	0.9433	0.9435	0.8608	0.9307	0.9439	0.8014	0.8341
page-blocks	0.9655	0.9677	0.9483	0.9667	**0.9688**	0.9627	0.9371	0.9673	0.9629	0.9455
penbased	0.9794	0.9456	0.9904	0.9567	0.9451	0.9942	0.9761	0.9478	0.9943	0.9904
phoneme	0.8247	0.8233	0.8562	0.8444	0.8261	0.8568	0.8142	0.8211	0.8555	0.8560
pima	**0.7620**	0.7292	0.6993	0.7330	0.7030	0.6717	0.7527	0.7318	0.6679	0.6980
ring	0.8746	0.8727	0.7174	0.9095	0.8846	0.9712	0.9177	0.8953	0.9691	0.7174
saheart	0.7189	0.6666	0.6685	0.6732	0.6906	0.5930	**0.7296**	0.6666	0.5864	0.6664
satimage	0.8642	0.8448	0.8907	0.8252	0.8483	0.8906	0.8738	0.8545	0.8883	**0.8940**
segment	0.9545	0.9385	0.9446	0.9160	0.9398	0.9597	0.9424	0.9437	0.9541	0.9446
sonar	0.7207	0.6688	0.7888	0.7014	0.6869	0.8269	0.7586	0.6914	0.7933	0.7888
spambase	0.9254	0.9056	0.8780	0.8951	0.9119	0.9250	0.9154	0.9089	0.9243	0.8780
spectfheart	**0.8023**	0.7752	0.7348	0.7370	0.7489	0.6860	0.6972	0.7604	0.7010	0.7500
splice	**0.9555**	0.9266	0.7210	0.8248	0.9276	0.9351	0.9273	0.9301	0.9379	0.7201
texture	0.9925	0.9015	0.9829	0.8500	0.9084	**0.9936**	0.9420	0.9124	0.9931	0.9831
thyroid	**0.9957**	0.9940	0.9161	0.9901	0.9949	0.9557	0.9526	0.9944	0.9543	0.9231
tic-tac-toe	**0.9666**	0.7829	0.8038	0.7035	0.7797	0.7734	0.7901	0.8006	0.7338	0.7975
titanic	0.7851	0.7874	0.6407	0.7837	0.7856	**0.7883**	0.7787	0.7842	**0.7883**	0.6407
twonorm	0.9764	0.8269	0.9454	0.8597	0.8358	0.9732	0.9722	0.8788	0.9745	0.9454
vehicle	**0.7874**	0.6926	0.6667	0.6030	0.6773	0.7576	0.6419	0.6809	0.7446	0.6619
vowel	0.7545	0.6566	0.8707	0.4556	0.6586	**0.8828**	0.6475	0.6586	0.8677	0.8535
wine	0.9549	0.8866	0.9330	0.7866	0.8987	0.9608	0.9605	0.8755	0.9719	0.9441
wisconsin	0.9564	0.9606	0.9623	0.9284	0.9417	0.9592	**0.9695**	0.9474	0.9547	0.9623
yeast	**0.5836**	0.5196	0.4967	0.4765	0.5472	0.5203	0.5512	0.5364	0.5176	0.5074
zoo	0.8917	0.8767	0.9331	0.7456	0.8875	0.7728	0.9117	0.9075	0.6822	0.9397

**Table 5:** Average rankings of the algorithms in 10% labeled ratio (Friedman) and Holm/Hochberg (alpha = 0.05).

Algorithm	Friedman ranking	*p* value	Holm/ Hochberg test
Self-LMT	6.8750	—	—
Tri-training (C45)	8.7019	1.5141*E* − 01	0.0500
Cobagging (C45)	9.2019	6.7671*E* − 02	0.0250
Co-Forest	9.6346	3.0238*E* − 02	0.0167
Democratic-Co	9.8558	1.9252*E* − 02	0.0125
Self-training (C45)	10.2308	8.4117*E* − 03	0.0100
Cotraining (SMO)	11.2115	6.6111*E* − 04	0.0083
SETRED	11.3846	3.9842*E* − 04	0.0071
Tri-training (SMO)	11.5000	2.8153*E* − 04	0.0063
SNNRCE	11.5000	2.8153*E* − 04	0.0056
Cotraining (C45)	11.5096	2.7340*E* − 04	0.0050
Cobagging (SMO)	11.5865	2.1587*E* − 04	0.0045
DE-tri-training (C45)	11.7788	1.1778*E* − 04	0.0042
Tri-training (NN)	11.9135	7.6088*E* − 05	0.0038
ADE-Co-Forest	12.0481	4.8633*E* − 05	0.0036
DE-tri-training (SMO)	12.1827	3.0754*E* − 05	0.0033
Self-training (SMO)	12.3173	1.9242*E* − 05	0.0031
Self-training (NN)	12.6827	5.1049*E* − 06	0.0029
APSSC	13.5769	1.4202*E* − 07	0.0028
CLCC	13.7500	6.7193*E* − 08	0.0026
Rel-Rasco (NB)	14.4231	3.0843*E* − 09	0.0025
Rasco (C45)	15.1346	8.8277*E* − 11	0.0024

**Table 6:** Average rankings of the algorithms in 20% labeled ratio (Friedman) and Holm/Hochberg (alpha = 0.05).

Algorithm	Friedman ranking	*p* value	Holm/ Hochberg test
Self-LMT	5.1923	—	—
Cobagging (C45)	8.7019	5.8533*E* − 03	0.0500
Tri-training (C45)	8.7692	4.9736*E* − 03	0.0250
Democratic-co	8.7788	4.8582*E* − 03	0.0167
Cotraining (C45)	9.5288	6.6111*E* − 04	0.0125
Co-Forest	9.7019	3.9842*E* − 04	0.0100
Self-training (C45)	10.0288	1.4596*E* − 04	0.0083
Cotraining (SMO)	10.2596	6.9191*E* − 05	0.0071
Cobagging (SMO)	10.4519	3.6267*E* − 05	0.0063
Tri-training (SMO)	10.8654	8.4002*E* − 06	0.0056
DE-tri-training (SMO)	11.1538	2.8515*E* − 06	0.0050
DE-tri-training (C45)	11.7404	2.7211*E* − 07	0.0045
Self-training (SMO)	12.0192	8.2869*E* − 08	0.0042
SETRED	12.0288	7.9474*E* − 08	0.0038
Self-training (NN)	12.7019	3.7052*E* − 09	0.0036
ADE-Co-Forest	12.8558	1.7697*E* − 09	0.0033
SNNRCE	12.9135	1.3364*E* − 09	0.0031
Tri-training (NN)	13.1538	4.0597*E* − 10	0.0029
APSSC	13.8462	1.0806*E* − 11	0.0028
CLCC	15.6058	2.9085*E* − 16	0.0026
Rel-Rasco (NB)	15.7019	1.5503*E* − 16	0.0025
Rasco (C45)	17.0000	1.8292*E* − 20	0.0024

**Table 7:** Average rankings of the algorithms in 30% labeled ratio (Friedman) and Holm/Hochberg (alpha = 0.05).

Algorithm	Friedman ranking	*p* value	Holm/ Hochberg test
Self-LMT	5.6923	—	—
Democratic-Co	8.8558	1.2989*E* − 02	0.0500
Cotraining (SMO)	9.4231	3.3946*E* − 03	0.0250
Cobagging (C45)	9.6923	1.6840*E* − 03	0.0167
Tri-training (C45)	9.7212	1.5583*E* − 03	0.0125
Tri-training (SMO)	10.2981	2.9846*E* − 04	0.0100
Co-Forest	10.3077	2.8988*E* − 04	0.0083
Cotraining (C45)	10.4423	1.9157*E* − 04	0.0071
Self-training (C45)	10.5096	1.5511*E* − 04	0.0063
Self-training (SMO)	10.6827	8.9048*E* − 05	0.0056
Cobagging (SMO)	10.7019	8.3632*E* − 05	0.0050
DE-tri-training (SMO)	10.8173	5.7133*E* − 05	0.0045
SETRED	12.3942	1.4202*E* − 07	0.0042
Self-training (NN)	12.7500	2.9907*E* − 08	0.0038
DE-tri-training (C45)	12.9135	1.4252*E* − 08	0.0036
ADE-Co-Forest	13.0577	7.3123*E* − 09	0.0033
Rasco (C45)	13.0962	6.1074*E* − 09	0.0031
SNNRCE	13.5288	7.5764*E* − 10	0.0029
Rel-Rasco (NB)	13.9135	1.0781*E* − 10	0.0028
Tri-training (NN)	14.0385	5.6118*E* − 11	0.0026
APSSC	14.2885	1.4781*E* − 11	0.0025
CLCC	15.8750	1.2868*E* − 15	0.0024

**Table 8:** Average rankings of the algorithms in 40% labeled ratio (Friedman) and Holm/Hochberg (alpha = 0.05).

Algorithm	Friedman ranking	*p* value	Holm/ Hochberg test
Self-LMT	5.0865	—	—
Democratic-Co	9.1923	1.2641*E* − 03	0.0500
Tri-training (C45)	9.2115	1.1990*E* − 03	0.0250
Cotraining (SMO)	9.4135	6.7962*E* − 04	0.0167
Cotraining (C45)	9.8942	1.5989*E* − 04	0.0125
Self-training (C45)	9.9519	1.3319*E* − 04	0.0100
Co-Forest	10.0577	9.4794*E* − 05	0.0083
Cobagging (SMO)	10.7212	9.6656*E* − 06	0.0071
Self-training (SMO)	10.7308	9.3332*E* − 06	0.0063
Tri-training (SMO)	10.8942	5.1049*E* − 06	0.0056
Cobagging (C45)	11.0096	3.3028*E* − 06	0.0050
DE-tri-training (SMO)	11.6923	2.1358*E* − 07	0.0045
DE-tri-training (C45)	12.1827	2.5157*E* − 08	0.0042
ADE-Co-Forest	12.2596	1.7753*E* − 08	0.0038
Rasco (C45)	12.2692	1.6992*E* − 08	0.0036
SETRED	12.3269	1.3048*E* − 08	0.0033
Self-training (NN)	13.0769	3.5107*E* − 10	0.0031
SNNRCE	13.5385	3.2060*E* − 11	0.0029
Tri-training (NN)	14.1827	9.1543*E* − 13	0.0028
APSSC	14.2404	6.5767*E* − 13	0.0026
Rel-Rasco (NB)	14.4808	1.6224*E* − 13	0.0025
CLCC	16.5865	1.7128*E* − 19	0.0024

## Appendix

A java software tool implementing the proposed algorithm and some basic run instructions can be found at http://www.math.upatras.gr/~sotos/SelfLMT-Experiment.zip.
